# Influence of Maternal Gestational Treatment with Mycobacterial Antigens on Postnatal Immunity in an Experimental Murine Model

**DOI:** 10.1371/journal.pone.0009699

**Published:** 2010-03-15

**Authors:** Muhammad Jubayer Rahman, Irene Roman Dégano, Mahavir Singh, Carmen Fernández

**Affiliations:** 1 Department of Immunology, Wenner-Gren Institute, Stockholm University, Stockholm, Sweden; 2 Lionex Diagnostics and Therapeutics GmbH, Braunschweig, Germany; Institut de Pharmacologie et de Biologie Structurale, France

## Abstract

**Background:**

It has been proposed that the immune system could be primed as early as during the fetal life and this might have an impact on postnatal vaccination. Therefore, we addressed in murine models whether gestational treatment with mycobacterial antigens could induce better immune responses in the postnatal life.

**Methods/Findings:**

BALB/c mice were treated subcutaneously (s.c.) at the second week of gestation with antigen (Ag)85A or heparin-binding hemagglutinin (HBHA) in the absence of adjuvant. Following birth, offspring mice were immunized intranasally (i.n.) with the same antigens formulated with the adjuvant cholera toxin (CT) at week 1 and week 4. One week after the last immunization, we assessed antigen-specific recall interferon gamma (IFN-γ) responses by *in vitro* restimulation of lung-derived lymphocytes. Protection against infection was assessed by challenge with high dose *Mycobacterium bovis* Bacille Calmette-Guérin (BCG) given i.n. We found that recall IFN-γ responses were higher in the offspring born to the treated mother compared to the untreated-mother. More importantly, we observed that the offspring born to the treated mother controlled infection better than the offspring born to the untreated mother. Since the gestational treatment was done in absence of adjuvant, essentially there was no antibody production observed in the pregnant mice and therefore no influence of maternal antibodies was expected. We hypothesized that the effect of maternal treatment with antigen on the offspring occurred due to antigen transportation through placenta. To trace the antigens, we conjugated fluorescent nanocrystals with Ag85A (Qdot-ITK-Ag85A). After inoculation in the pregnant mice, Qdot-ITK-Ag85A conjugates were detected in the liver, spleen of pregnant females and in all the fetuses and placentas examined.

**Conclusion:**

The fetal immune system could be primed *in utero* by mycobacterial antigens transported through the placenta.

## Introduction

*Mycobacterium bovis* Bacille Calmette-Guérin (BCG) has been used widely as a vaccine against tuberculosis (TB) since 1921. Unfortunately, failure of BCG vaccination has been reported due to poor and variable efficacy against adult pulmonary TB [1]. Apart from these limitations, neonatal BCG vaccination has a number of adverse effects, i.e. systemic BCGosis, a rare but severe consequence of BCG vaccination in children with immune deficiencies or HIV infection [2]. Consequently, the development of an effective vaccine, either a modified form of BCG or a replacement of BCG vaccine suitable for the neonatal period of vaccination is of utmost importance.

In general, an effective vaccination of newborns represents a major challenge; given the fact that the neonatal immune system is not fully matured and therefore not ready to respond to vaccination in an efficient manner. Neonatal T cell responses are suboptimal both qualitatively and quantitatively as suggested by Sarzotti *et al* [3]. Compared to the adults, neonates are reported to be aberrant in their cellular immune responses, often biased to develop Th2 type immune responses and decreased ability to generate Th1 type immune responses [4], [5]. However, it has been proposed that neonatal T cells from both humans and mice, are able to mount a mature Th1 response provided that the magnitude of costimulatory signals is increased [reviewed in ref. 5]. BCG vaccine is one of the classic examples that can alter the unresponsiveness of the neonatal immune system upon vaccination, resulting in a robust Th1 type of immune response [6], [7]. Moreover, vaccines formulated with the strong Th1-promoting agent CpG motif or IC31 adjuvant [6], [8] or vaccines delivered via mucosal route could impact on early-life vaccination strategies [9], [10].

Studies in both murine and human models proposed that the onset of sensitisation of the fetal immune system might be possible *in utero* [11]–[13]. In humans, prenatal exposure to helminth or mycobacterial antigens has been shown to lead to better cytokine responses in neonates [11]. Also, a memory T cell phenotype was observed upon restimulation *in vitro* of cord blood mononuclear cells with various allergens suggesting that T cell priming might occur *in utero* [14]. Of interest is that depending on the conditions of antigen exposure, neonates could respond differently ranging from deficient to fully matured forms of immune responses [reviewed in ref. 6]. However, even if it is suggested that *in utero* sensitization may occur due to transplacental transfer of antigens [15], it is still uncertain how the fetal immune system gets primed with the antigen.

The aim of our study was to understand how the prenatal sensitisation occurs and its impact on the development of a new TB vaccine. We have tested two mycobacterial protein antigens namely antigen (Ag)85A and the native form of mycobacterial heparin-binding hemagglutinin (nHBHA) for immunization. Both nHBHA and Ag85A have been evaluated as TB vaccine candidates in adult animal models [16]–[19]. It has been shown that only native but not recombinant (r) HBHA could induce protective immune responses [16]. Ag85A is one of the major secretory proteins that has been tested in clinical trials as a booster vaccine and reported to be safe and immunogenic [20].

Our hypothesis was that maternal gestational treatment with antigen could result in priming of the fetal immune system and that subsequent immunization during the postnatal life may increase neonatal immunity. We reported herein that exposure to mycobacterial antigens during the gestational period leads to antigen transportation from the mother to the fetus and this resulted in an early priming of the fetal immune system. Immunization with the same antigen during the postnatal life increased antigen-specific recall interferon gamma (IFN-γ) responses and protection against infection.

## Results

### Maternal treatment with antigen increases postnatal cellular immune responses but shows no effect on humoral immune responses

To follow the antigen-priming effect from the fetal age, we treated a group of pregnant mice at the 2^nd^ week of pregnancy with Ag85A given alone via subcutaneously (s.c.). Following birth, offspring mice were immunized twice at week 1 and week 4 with the same antigen plus adjuvant administered intranasally (i.n.). One week after the last immunization, lung lymphocytes were isolated and restimulated *in vitro* with Ag85A in order to assess the recall IFN-γ responses in the culture supernatants. As shown in Table 1, offspring born to the treated mother had significantly higher recall immune responses to Ag85A than the offspring born to the untreated mother (Group 1 versus Group 3). In contrast, maternal treatment did not show any significant influence on serum antibody responses in the offspring.

**Table 1:** Effect of prenatal treatment on postnatal immune responses.

Prenatal			Postnatal					
	Groups	Gestational treatment^a^	Immunization (2×)^b^	Nursing mother^c^		Immune responses		
						IFN-γ (pg/ml)	IgM (O.D.)	IgG (O.D.)
Exp. 1	1	+	+		Ag+	1694±167*	0.73±0.05	1.01±0.04
	2	+	+		Ag−	1736±24*	0.67±0.03	0.86±0.12
	3	-	+		Ag−	1045±30	0.63±0.06	0.78±0.06
Exp. 2	1	+	+		Ag+	3358±79*	0.54±0.17	0.44±0.09
	2	+	+		Ag−	3308±616*	0.68±0.22	0.32±0.03
	3	-	+		Ag−	1866±499	0.52±0.06	0.40±0.04

a Pregnant mother at the 2^nd^ week of gestation received Ag85A administered via s.c.

b Offspring mice were immunized twice with Ag85A at week 1 and week 4.

c Offspring mice were breastfed by treated (Ag+) or untreated (Ag−) mother immediately after birth until the weaning period.

O.D. values represent mean±s.d. of three mice with 1∶1600 dilution.

Data for IFN-γ represent mean±s.d. of triplicates prepared from pooled samples (3–4 mice/group).

*, p<0.05 when Group 1 or 2 compared with Group 3.

In order to investigate whether the breast milk of a treated mother had any influence on postnatal immune responses, a group of newborn mice born to the treated mother was nursed by a foster mother (Table 1, Group 2) until the weaning period. Results showed that gestational priming enhanced recall responses regardless of the condition of postnatal nursing (Table 1, Group 2), which were comparable to the group nursed by their own mother (Table 1, Group 1). Antigen-specific IgM and IgG levels in serum samples were similar between the groups. Neither cytokine levels nor antibody levels were significantly enhanced by postnatal nursing delivered by the treated mother (data not shown).

Finally, we checked the immune responses in a group of adult mice treated similarly to the pregnant mothers. We observed that treatment with the antigen in absence of adjuvant did not induce any detectable IgG responses (Fig. S1). To induce a detectable IgG response, Ag85A had to be provided together with the adjuvant (Fig. S1).

### Transplacental transfer of antigen from the mother to the fetus

**Figure 1:**
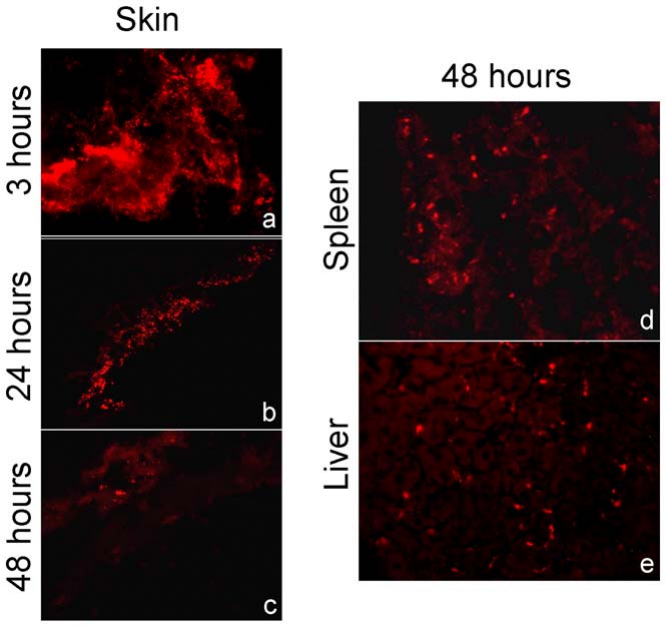
In vivo Qdot distribution. Adult mice (3 animals per group) were injected with Qdot-ITK (120 pmol/mouse) via s.c. Mice were sacrificed at several time points (3 h, 24 h and 48 h) post-injection and skin, kidneys, liver, lungs and spleen were collected and embedded in OCT. Sections were fixed, mounted and observed under green light in a fluorescence microscope. Qdot-ITK fluorescence was detected in skin slides at 3 h (a), 24 h (b) and 48 h (c) post inoculation, and in slides from the spleen (d) and the liver (e) at 48 h post inoculation. Magnification 100×.

Prior to examine the translocation of antigens by injecting Qdot-ITK-Ag85A to pregnant mothers, we injected plain Qdot-ITK s.c. and followed their *in vivo* distribution. Qdot-ITK were detected mainly in the skin, liver, and spleen. Qdot-ITK fluorescence in the skin decreased with time (Fig. 1a, b, c). In the spleen and liver, fluorescence signals increased from 24 h to 48 h post injection (Fig. 1d, e).

**Figure 2:**
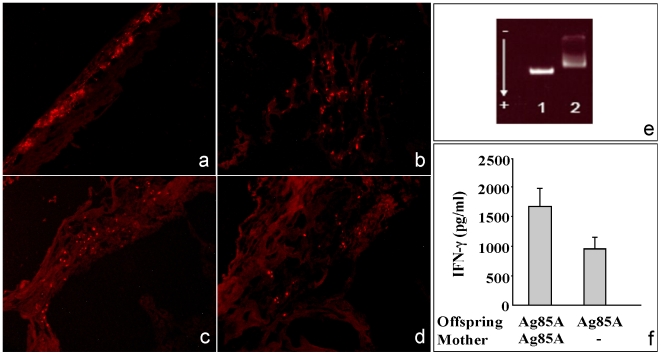
Distribution of Qdot-ITK-Ag85A in mother and fetuses. Pregnant mice (3 animals per group) were injected with Qdot-ITK-Ag85A (120 pmol/mouse) via s.c. Mice were sacrificed 48 h post inoculation and mother organs as well as fetuses and placentas were removed and embedded in OCT. Sections were fixed, mounted and observed under green light in a fluorescence microscope. Qdot-ITK-Ag85A fluorescence was detected in the skin (a), placenta (b) and fetuses (c, d). Magnification 100×. Prior to the inoculation, Qdot-ITK-Ag85A were run in a 0.5% agarose gel (e) to check conjugation. Lane 1, unconjugated Qdot-ITK; lane 2, Qdot-ITK-Ag85A. Recall IFN-γ responses after *in vitro* restimulation of lung cells with Ag85A were measured in the offspring born to the mother that received Qdot-ITK-Ag85A or Qdot-ITK (f).

To follow the transfer of antigen from the mother to the fetus, we injected the conjugate Qdot-ITK-Ag85A in pregnant mice at day 11 of gestation. Forty-eight hours postinjection, tissue specimens were collected and fluorescence signals were detected. As shown in Fig. 2, Qdot-ITK fluorescence was detected in maternal organs and in all placentas and fetuses analyzed. (Fig. 2c, d).

To check whether treatment with Qdot-ITK-Ag85A resulted in priming of the fetal immune system, separate groups of pregnant mice were allowed to give birth and postnatal immunization was performed as described above. Similarly, Ag85A-specific recall IFN-γ responses were also increased in the offspring mice born to the Qdot-ITK-Ag85A-treated mother (Fig. 2f).

### Gestational treatment leads to higher levels of protection against mycobacterial infection in the postnatal age

**Figure 3:**
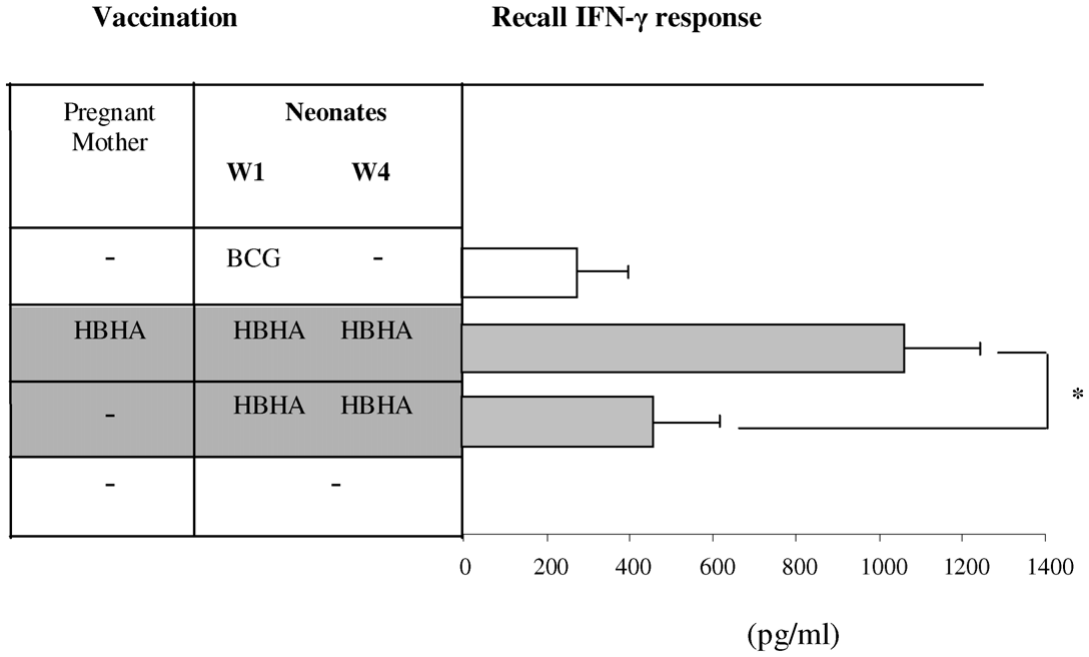
Maternal treatment increases postnatal cellular immune responses. Pregnant mice at 2^nd^ week of gestation were treated with rHBHA via s.c. Following birth, neonates were immunized twice i.n. with rHBHA formulated with CT at week 1 (W1) and week 4 (W4). One week after the last immunization, lung cells from the rHBHA-immunized animals were re-stimulated *in vitro* with rHBHA to measure recall IFN-γ responses. Data show mean IFN-γ ± s.d. of triplicate wells prepared from pooled samples (3–4 mice/group). Results were analyzed from two independent experiments. p value (s) was calculated by comparing two groups using *t* test. * significant when p<0.05.

In order to investigate whether the increased postnatal responses could have an impact on protection against infection later in life, we chose mycobacterial nHBHA for immunization of mice. nHBHA is one of the few antigens that has been tested in animal models and found to provide a level of protection similar to that seen after BCG vaccination [16]. Although rHBHA is not a protective antigen, upon immunization, both forms of HBHA could induce a comparable level of immune responses *in vitro* [16]. Thus, to assess antigen-specific memory responses, mice were immunized with rHBHA and antigen-specific recall immune responses were determined. Results from this study showed that like Ag85A, prenatal treatment with rHBHA could induce higher immune responses in the offspring compared to the control group born to the mother untreated gestationally (Fig. 3).

To examine the level of protection, separate groups of mice vaccinated with nHBHA were challenged with a high dose of BCG (10^7^ CFU) given i.n., and 3 weeks postchallenge the bacterial burden in the lungs was determined. As shown in Table 2, the offspring born to the treated mother received nHBHA after birth could control infection better than the offspring born to the untreated mother (Group 2 versus Group 3, p<0.01). The effect of maternal priming was less clear in the group that received BCG instead of nHBHA at 1-week and boosted later with nHBHA (data not shown).

**Table 2:** Bacterial burden in the lungs of offspring mice born to the mother treated with or without nHBHA.

Treatment/Vaccination			Protection	
Groups	Pregnant Mother	Neonates	CFU in the lungs mean±sd ×10^4^	% of reduction
		Week 1 Week 4		
1	-	BCG -	3.95±0.6	21
2	nHBHA	nHBHA+CT nHBHA+CT	3.15±0.7	37**
3	-	nHBHA+CT nHBHA+CT	4.40±0.6	12
4	-	-	5.00±0.7	-

Data show bacterial counts from individual animals and the mean. Four animals per group from two independent experiments are depicted. Values shown are the percent of reduction of CFU, calculated with respect to the PBS-control group.

**, p<0.01 when Group 2 versus Group 3.

Not significant when Group 1 versus Group 2.

## Discussion

Even if certain vaccination protocols can provide protection during the neonatal period, frequently, successful vaccination is compromised by the poorly developed immune system in neonates [21]. This is unfortunate and leaves newborns exposed to infection during a sensitive period in their lives. To improve this situation, two strategies have been proposed namely, immunization of the pregnant mother allowing the transfer of specific antibodies through the placenta and milk and early priming of the fetal immune system by *in utero* exposure to foreign substances. The relevance of the first strategy is widely accepted [22], [23] but it has been questioned whether the fetal immune system could be primed by simple exposure to the antigens or if various maternal immune factors could contribute to the priming effect. The aim of the present study has been to investigate this issue.

In this study we have shown that mycobacterial antigens administered to the mother during the 2^nd^ week of gestation were transported to the fetus through the placenta and that offspring born to the treated mother displayed higher specific T cell responses compared to the offspring born to the untreated mother. Moreover, this first group of mice was more efficient in controlling bacterial growth upon i.n. infection with BCG.

The maternal treatment was done under suboptimal immunization conditions since the antigens were administered in the absence of adjuvant. As expected, no specific antibodies could be observed in the serum of the treated animals unless the antigen was given twice and formulated together with CT as adjuvant. Under these conditions, protection in the offspring may be attributed more to the transfer of antigen than to the transfer of antibodies from the mother to the fetus. In addition of having antigens transferred through the placenta, maternal gestational treatment could also result in antigen transfer via mother's milk [24]. In this study, we did not directly monitor antigen transfer from the mother to the neonates through the milk, however, antigen-specific recall responses in the offspring mice were not affected due to postnatal nursing provided by foster mothers (untreated).

Data from our study clearly showed that protein antigens were transported from the mother to the fetus through placenta since antigens conjugated with fluorescent Qdot were visible on the placental tissue as well as on the fetal tissue. However, we do not have any data about the organ distribution of antigen in the fetus. This may be worth to consider for future studies. Our findings corroborate earlier observations in humans where it was postulated that prenatal exposure to antigens alters fetal immunity [11], [25]–[27]. However, in those studies, it was not possible to evaluate the effect of antigen alone since the long period of human pregnancy complicated the issue. The immune response of the mother and the influence of transfer of specific antibodies through the placenta or by milk could not be disregarded.

We observed here only a priming of the T cell compartment. In mice and rats, the appearance of B cells is delayed compared to the T cells and no B cells have been observed before birth. Demarcation of splenic architecture takes place by day 6 and germinal centers are observed between days 21 and 28 after birth. In contrast, fetal thymocytes are able to proliferate in response to T cell mitogens by day 17 of gestation [28]. This may explain why only T cell and no B cell priming effect was observed upon exposure to antigen delivered to the pregnant mothers at week two of the gestation time. T cell development is completed in mice immediately before birth and only a few mature CD4 and CD8 T cells appear during this time which makes it difficult to analyze the type of memory cells developed after gestational treatment [29]. In humans, small numbers of B and T cells are already present in the fetal liver and the architecture of the spleen is demarcated by week 26 of gestation. Thus, before birth, humans have relatively well functional B and T cells which is not the case in mice and rats [28]. Consequently, priming of human fetal B cells may be achieved by exposure to antigen: certain evidence has already been provided as in the case of maternal immunization with tetanus toxoid during pregnancy [26]. Also, the priming of human T cells *in utero* has been demonstrated since cord-blood cells from PPD-sensitized mothers produced more antigen-specific cytokines compared to cord-blood from unsensitized mothers [11].

We can conclude that gestational priming favoured the induction of Th1 type responses in mice as evident by the increased levels of IFN-γ and a better control of infection, which was comparable to the BCG-vaccinated control group. Thus, our observations support, at least for these two mycobacterial protein antigens, that prenatal immunization can sensitize the fetal immune system by transplacental transfer of antigens. Consequently, antigen specific T cell responses were increased following postnatal immunization suggesting that this approach does not induce tolerance in the neonates. Recently, Malhotra I *et al* demonstrated that prenatal exposure to helminth and mycobacterial antigens does not induce tolerance but could enhance cytokine responses in human fetus [11].

The basic significance of this current study lies on the fact that prenatal immunization might impact on the maternal-fetal interaction and such manipulation could contribute to the subsequent responses to postnatal immunization. The critical factor might be the time point of gestational priming, which has been described in several previous studies [24], [26], [30]. In humans, it has been proposed that a cut-off level greater than 22 weeks gestation favors *in utero* sensitisation with allergens [30]. Similarly, Gill TJI *et al* suggested that transplacental immunization of human fetus occurs during fifth to eight months of pregnancy [26]. This approach might provide new insights into the development of TB vaccines especially for the people who are at risk of adverse situation upon BCG-vaccination.

Another aspect of importance may be the route of antigen administration. Subcutaneous delivery of the antigen may be crucial due to the presence of various types of professional antigen presenting cells such as DCs and Langerhans cells in the region [31]. These cells may be responsible for the transport and efficient presentation of the antigen from the mother to the fetal immune system. In this study, offspring mice were immunized via i.n. in order to induce a better protection in the respiratory tract. *Mycobacterium* has a preferential tropism for the lungs and in fact, tuberculosis is primarily a lung disease. We and others have shown that i.n. immunization is better than systemic immunization to evoke protective immune responses in the respiratory tract [32], [33]. Thus, we considered more important to study immune responses and bacterial growth after infection in the lungs.

We conclude that prenatal priming of the fetal immune system via transplacental transfer of antigens is possible and that this treatment could educate the immune system of the neonates to be ready for vaccination immediately after birth. This way of vaccination might contribute significantly to the strategy being used currently towards the development of new vaccines and perhaps meet the needs for a successful neonatal vaccination.

## Materials and Methods

### Mice

The studies were performed using adult (10 weeks), neonatal (1 week) and infant (4 to 6-weeks) BALB/c mice. Adult mice were purchased from Taconic Europe, Denmark or Scanbur AB, Sweden and housed in pathogen free conditions. Neonates were obtained from laboratory breeding facilities followed by timed (hand) mating (for 24 hours) protocol using adult males and females manually placing them together for an over night period. Three weeks after birth, mice were weaned and separated from their mothers. All animals were kept at the Animal Department of the Arrhenius Laboratories, Stockholm University, Sweden. All experiments were done in accordance with the ethical guidelines available at Stockholm University. Mice were supervised daily and sentinel mice were used to assess and ensure pathogen free conditions in the facility.

### Antigens and adjuvants

Recombinant Ag85A was obtained from LIONEX Diagnostics & Therapeutics GmbH, Germany. Both n- and rHBHA were kindly provided by C. Locht, Pasteur de Lille, France. Briefly, nHBHA was extracted from *Mycobacterium tuberculosis or M. bovis* and purified by heparin-sepharose chromatography [34], followed by high-performance liquid chromatography (HPLC) as described previously by Masungi *et al* [35]. rHBHA was expressed in *Escherichia coli* and purified by nickel chromatography as previously described [36]. Adjuvant, cholera toxin (CT) was obtained from Quadratech Ltd, Surrey, UK.

### Mycobacteria

*M. bovis* BCG (Pasteur strain) obtained from A. Williams, HPA, Salisbury, UK was grown in Middlebrook 7H9 (DIFCO, Sparks, MD, USA) broth supplemented with albumin-dextrose-catalase (ADC), 0.5% glycerol and 0.05% Tween 80 (vol/vol). BCG was collected at a log phase of growth (absorbance 1.0 measured at OD_600_) for a culture period of 10–15 days at 37°C. Aliquots were frozen in phosphate buffer saline (PBS) with 10% glycerol and kept at −70°C. Three vials picked randomly from the stock were thawed, serially diluted in plating buffer (PBS with 0.05% Tween-80 [vol/vol]) and colony forming units (CFU) counted 2–3 weeks after plating on Middlebrook 7H11 agar (Karolinska Hospital, Solna, Sweden) prepared with glycerol, oleic acid-albumin-dextrose-catalase (OADC) and antibiotics; polymyxin B and amphotericin B.

### Immunization

As shown in Table 3, pregnant mice 14 days post mating (2^nd^ week of gestation) were treated once with Ag85A or HBHA (10 µg/mouse) formulated in PBS administered s.c. at the dorsal neck region. A control group of pregnant mice received PBS. After birth, mice were immunized twice i.n. with Ag85A or HBHA, at week 1 (2 µg Ag85A or 1 µg HBHA formulated with 0.2 µg CT in 5 µl of PBS/mouse) and week 4 (10 µg Ag85A or 5 µg HBHA formulated with 1 µg CT in 20 µl of PBS/mouse). A group of newborn mice from Ag85A-treated mother was nursed by the PBS-treated mother and vice versa (Table 3). One week old mice received a dose which was five-times lower than that prepared for 4 weeks old mice. Mice were anesthetized with isofluorane (Baxter Medical AB, Kista, Sweden) and 5 µl of the formulation was delivered per nostril for a total of two doses. Mice were allowed to breathe the suspension into the lungs naturally. Immunization at week 1 was performed without the use of isofluorane. One week after the last immunization, mice were sacrificed and blood and lungs were collected.

**Table 3:** Immunization schedule.

Treatment^1^		Nursing Mother^2^
Pregnant mice	Offspring mice (2X)	
+	+	Ag+
-	+	Ag−
+	+	Ag−
-	+	Ag+
-	-	Ag−

1 Pregnant mice were treated with Ag85A or HBHA at the 2^nd^ week of gestation. Offspring mice born to the mothers were immunized with Ag85A or HBHA at week 1 and week 4 during postnatal age.

2 Offspring mice were nursed by either treated (Ag+) or untreated (Ag−) mother until week 3.

To assess for the lack of antibody production by the antigen-treated pregnant females, a separate group of adult mice was given Ag85A without adjuvant. Primary and secondary specific antibody responses were monitored in the serum up to week 2 for the primary responses and one week after the last immunization for the secondary responses. The immunogenicity of Ag85A was evaluated when the antigen was provided in the presence of CT as adjuvant. Secondary responses were measured also one week after the last immunization.

### Infection and bacterial counts in organs

Four weeks after the last immunization of the offspring with nHBHA, mice were challenged with a high BCG dose (10^7^ CFU) i.n. Three weeks after infection, lungs were removed aseptically. The organs were homogenized by using a glass homogenizer. Organ homogenates were diluted serially and plated on Middlebrook 7H11 agar plate and incubated at 37°C for 2–3 weeks. Following incubation, bacterial counts were enumerated and expressed as CFU per organ.

### Quantum dot conjugation and in vivo administration

Poly-ethylene-glycol coated quantum dots (Qdot-ITK) that emit light at 655 nm were purchased from Invitrogen and stored at 4°C. Qdot-ITK were coupled to Ag85A following a previously described protocol [37]. Briefly, 400 pmol of Qdot-ITK were mixed with 16 nmol of Ag85A and 1.6 µmol of EDC (N-(3-dimethyl-aminopropyl)-N'-ethyl-carbodimide hydrochloride) (Sigma, St. Louis, MO, USA) and the pH was adjusted to 7.4 and incubated for 1 h at room temperature. Nanosep spin filters of 100-kilodalton cutoff (Pall, East Hills, NY, USA) were washed first with 100 µl of 1 mM NTA (N,N-bis-(carboxymethyl)-L-lysine hydrate) (Sigma) and secondly with 500 µl of Tris HCl buffer. After washing, the conjugate was collected and stored at 4°C until use. To check if conjugation had taken place and conjugate fluorescence, Qdot-ITK and conjugated Qdot-ITK-Ag85A were separated by a 0.5% agarose gel and visualized using ultraviolet light (Fig. 2e).

Due to ethical reasons, pregnant mice (3 per group) at day 11 were anesthetized with isofluorane and 100 µl of solution containing 120–130 pmols of Qdot-ITK or Qdot-ITK-Ag85A were injected s.c. We reasoned that there would be no major differences in placental transfer between day 11 and day 14 of gestation since mouse placenta from day 8.5 to day 10.5 is known to be ready to exchange materials from the mother to the fetus [38]. Also placenta examined from day 10 to day 18 of pregnancy revealed that cells important for building three layers: the labyrinth, the spongiotrophoblast, and the maternal deciduas, are present during this period although variations in the number and function of cells could be expected [39]. Mice were sacrificed at 3, 24 and 48 h post-injection by cervical dislocation and skin, kidneys, liver, lungs, and spleen as well as placentas from pregnant females and whole fetuses were collected, washed with PBS and embedded in OCT compound (Sakura, IMEB, San Marcos, CA, USA). Sections (10 µm) were prepared using a cryostat (Leica, Wetzlar, Germany) and fixed at 4°C with cold acetone, washed in distilled water and mounted with Neo-Mount (Merck, Darmstadt, Germany). The slides were observed with a fluorescence microscope (Reichert Microscope, Depew, NY, USA) using green light to visualize Qdot-ITK or Qdot-ITK-Ag85A conjugates.

### Mononuclear cell isolation

One week after the last immunization with Ag85A or rHBHA, lungs were collected in sterile PBS, cut into small pieces (2–3 mm) and incubated for one hour at 37°C with collagenase type I (1 mg/ml, Roche, Mannheim, Germany) and DNase (30 U/ml, Sigma). Lung pieces were then crushed by using a glass homogenizer and the cell suspension was passed through a 70 µm nylon membrane (BD, Franklin Lakes, NJ USA) and subjected to gradient separation using Lympholyte M (CEDARLANE laboratories, Ontario, Canada). After centrifugation at 1500 g for 20 minutes, the interface was collected and washed twice with PBS. Viable cells were enumerated using trypan blue. Thereafter, cells were cultured in complete DMEM medium containing 10% fetal calf serum, 2 mM L-glutamine, 100 U/ml penicillin, 100 µg/ml streptomycin and 2 mM sodium pyruvate (all from Invitrogen, Paisley, UK).

### In vitro Ag-restimulation of lymphocytes

Mononuclear cells were isolated from the lungs and plated (2×10^5^ cells/well) in 96-well flat bottom plates (Costar, NY, USA) and stimulated with rHBHA (5 µg/ml), Ag85A (10 µg/ml) or Con A (2 µg/ml) for 72 h at 37°C, 5% CO_2_. Cell culture supernatant was collected and stored at −20°C until tested for cytokines by Enzyme-linked immuno sorbent assay (ELISA) assay.

### IFN-γ ELISA

A commercially available kit for IFN-γ (Mabtech, Stockholm, Sweden) was used to determine the cytokine levels in the culture supernatants according to the manufacturer's recommendations, with slight modifications. Streptavidin conjugated to alkaline phosphatase was used instead of horseradish peroxidase at 1∶1000 dilution. The enzyme-substrate reaction was developed using p-nitrophenyl phosphate (Sigma). Optical density (O.D.) was measured in a multiscan ELISA reader at 405 nm and concentrations were calculated from the standard curves established with corresponding purified recombinant mouse IFN-γ. Data are presented as pg/ml concentration.

### Detection of antibodies in serum

Antibodies in serum were measured by ELISA. High-binding ELISA plates (Costar, high binding, NY, USA) were coated with Ag85A (2 µg/ml) in carbonate-bicarbonate buffer (pH 9.6) and incubated overnight (ON) at room temperature (RT). Plates were washed four times with washing buffer (0.9% NaCl- 0.05% Tween-20). After washing, samples from individual animals from each group (3–4 mice/group) were serially diluted with a starting dilution 1∶200 and added to the antigen coated plates and then incubated ON at RT. Following sample incubation, plates were washed and incubated for 2 h at RT with alkaline-phosphatase (ALP) labelled goat anti-mouse IgM or IgG (Southern Biotech, Birmingham, USA), and the enzyme substrate reaction was developed using p-nitrophenyl phosphate as substrate. Optical density was measured in a multiscan reader at 405 nm.

### Statistical analysis

Comparison between two experimental groups was done by Student's *t* test. *p* values of <0.05, and <0.01 were considered as the levels of significance.

## Supporting Information

Figure S1 Secondary antibody levels after priming with Ag85A. Adult mice (3 animals per group) were immunized with Ag85A formulated with or without CT in a 2- weeks interval. Serum samples were collected after primary week 1 (W1) and week 2 (W2) and secondary week 1 (W1) immunizations. Antigen-specific IgG levels were determined by ELISA. Data showed mean O.D. ± s.d. of antibodies in serum of 3 mice. Broken line indicates the cut-off level after primary immunization with or without CT.
(0.03 MB DOC)
