# Supplementary material for: Influence of Maternal Gestational Treatment with Mycobacterial Antigens on Postnatal Immunity in an Experimental Murine Model
Source: PLoS One. 2010 Mar 15;5(3):e9699. doi: 10.1371/journal.pone.0009699 (PMC2837747; doi:10.1371/journal.pone.0009699)
Supplement: Figure S1 — Secondary antibody levels after priming with Ag85A. Adult mice (3 animals per group) were immunized with Ag85A formulated with or without CT in a 2- weeks interval. Serum samples were collected after primary week 1 (W1) and week 2 (W2) and secondary week 1 (W1) immunizations. Antigen-specific IgG levels were determined by ELISA. Data showed mean O.D. ± s.d. of antibodies in serum of 3 mice. Broken line indicates the cut-off level after primary immunization with or without CT. (0.03 MB DOC) [file pone.0009699.s001.doc]

O.D (405nm)

**Figure S1**

0

0,1

0,2

0,3

0,4

0,5

0,6

0,7

0,8

0,9

1

+ CT

- CT

W0 W1 W2

**Primary**

**Secondary**

- /+ +/+ CT
